# Assessment of Nuclear ZEB2 as a Biomarker for Colorectal Cancer Outcome and TNM Risk Stratification

**DOI:** 10.1001/jamanetworkopen.2018.3115

**Published:** 2018-10-05

**Authors:** Rahul Sreekumar, Scott Harris, Karwan Moutasim, Ricardo DeMateos, Ashish Patel, Katherine Emo, Sophie White, Tamer Yagci, Eugene Tulchinsky, Gareth Thomas, John N. Primrose, A. Emre Sayan, Alex H. Mirnezami

**Affiliations:** 1 Cancer Sciences Division, Faculty of Medicine, University of Southampton, Southampton, United Kingdom; 2 Department of Surgery, Southampton University Hospital National Health Service Trust, Southampton, United Kingdom; 3 Medical Statistics and Mathematics Department, University of Southampton, Southampton, United Kingdom; 4 Department of Medical Oncology, Dana-Farber Cancer Institute, Harvard Medical School, Boston, Massachusetts; 5 Department of Molecular Biology and Genetics, Gebze Technical University, Gebze, Turkey; 6 Department of Cancer Sciences, University of Leicester, Leicester, United Kingdom

## Abstract

**Importance:**

At present, patients with colorectal cancer (CRC) are risk stratified using TNM histologic features. More recently, an association between a mesenchymal phenotype and a high risk of disease recurrence and micrometastases has been recognized.

**Objective:**

To investigate the association of the epithelial to mesenchymal transition (EMT)–inducing transcription factor ZEB2 (zinc finger E box–binding homeobox 2), survival outcomes, and the efficacy of ZEB2 as a biomarker when added as refinement to TNM staging after curative intent surgery for CRC.

**Design, Setting, and Participants:**

ZEB2 expression was assessed using a previously validated scoring system as part of a prospective, observational, masked diagnostic study from January 1, 2008, to December 31, 2013. Data were prospectively collected and analyzed for association with oncologic outcomes from January 1, 2017, to December 31, 2018. An initial test cohort from an academic university medical center of 126 consecutive patients with CRC and, subsequently, an independent validation cohort of 210 patients were examined. ZEB2 positivity was scored by 2 independent, masked pathologists. External validity was tested using an open access gene expression portal. Nomograms were developed with or without ZEB2.

**Main Outcomes and Measures:**

Systemic and local recurrence of CRC.

**Results:**

The test cohort consisted of 126 consecutive patients (mean [SD] age, 72.7 [11.7] years; 61 [48.4%] male) and the validation cohort of 210 patients (mean [SD] age, 72.0 [10.6] years; 111 [52.9%] male). A total of 52 tumors (41.3%) in the test cohort and 104 (49.5%) in the validation cohort were scored nuclear ZEB2 positive. Survival analysis by the log-rank test found that ZEB2 expression was associated with a significant reduction in overall survival and disease-free survival in both cohorts. Cox proportional hazards regression analysis highlighted ZEB2 as an independent biomarker of shorter overall survival and disease-free survival. Analysis of node-negative disease (n = 222) identified ZEB2 as an independent biomarker of early recurrence and reduced survival. External validation confirmed these findings. Addition of ZEB2 expression to nomograms composed of conventional TNM risk factors improved the ability to identify patients at high risk of recurrence demonstrated by the improvement in concordance index in both test (0.73 to 0.77) and validation (0.82 to 0.87) cohorts.

**Conclusions and Relevance:**

The findings suggest that expression of ZEB2 is associated with poor oncologic outcome and distant recurrence. The study also found that the addition of ZEB2 to existing TNM classification improved the ability to stratify patients for risk of recurrence. The results of this study suggest that addition of ZEB2 expression status to the TNM staging system improves the ability to stratify patients at high risk of recurrence.

## Key Points

**Question:**

What is the clinical utility of the epithelial to mesenchymal transition program in assessing outcome in patients with colorectal cancer?

**Findings:**

In this diagnostic study with validation in 2 independent cohorts of 336 individuals, expression of the epithelial to mesenchymal transition inducing transcription factor ZEB2 was associated with poor oncologic outcomes and improved ability to risk stratify patients when added to the TNM classification, potentially affecting 14 of 100 patients.

**Meaning:**

The findings suggest that epithelial to mesenchymal transcription factor ZEB2 is a biomarker with clinical potential and may improve TNM risk stratification and guide treatment in colorectal cancer.

## Teaser

This diagnostic study investigates whether nuclear ZEB2 expression in colorectal cancer is associated with early recurrence and reduced survival in 2 independent patient cohorts.

## Introduction

Colorectal cancer (CRC) is the most common gastrointestinal cancer in Western countries.^1^ Surgery remains the mainstay of treatment; however, metachronous systemic and local recurrence from occult micrometastatic spread is common and the principal cause of mortality.^2^ One method to reduce recurrence risk is by modern combination adjuvant chemotherapy. Nevertheless, decision-making on application of adjuvant chemotherapy is challenging and inexact. As a result, many patients are overtreated with adjuvant chemotherapy and exposed to its detrimental effects, whereas others at risk of early-stage disease are not offered adjuvant chemotherapy but develop recurrence from undertreated micrometastases. These observations underscore the imprecise methods for staging, identification of micrometastases, and selection for adjuvant chemotherapy and highlight the critical need for better markers to identify occult spread.^3^

For decades, the TNM staging system, based on depth of tumor invasion and lymphatic node or distant organ spread, has been used to stratify patient risk and determine the need for adjuvant chemotherapy.^4^ More recently, there has been increasing acknowledgment of the limitations of the TNM staging system and its failure to incorporate novel biological markers that may better inform disease trajectory.^5^ Consequently, refinement of the TNM staging system through addition of validated molecular biomarkers of micrometastatic spread is an attractive concept and may improve recognition of patients whose conditions are currently erroneously understaged or overstaged.

Epithelial to mesenchymal transition (EMT) is a conserved epigenetic program that generates motile invasive mesenchymal cells from epithelial sheets under physiologic conditions.^6^ In cancer, aberrant activation of this molecular program endows cancer cells with enhanced metastatic properties. Epithelial to mesenchymal transition is activated by signaling pathways that upregulate expression of EMT-inducing transcription factors.^7^ In CRC, numerous in vitro and preclinical studies^8,9,10^ have found that EMT leads to increased metastatic capacity and apoptosis resistance to commonly used chemotherapeutic agents. An association between EMT and poor oncologic outcomes has also been reported in other solid organ malignant tumors.^9,11,12,13^ More recently, high-profile independent studies^14,15,16,17^ have conducted molecular profiling of CRC, and although they have differed in the number of subtypes identified, tumors with an EMT-mesenchymal signature are consistently associated with poor outcomes. Nevertheless, the presence of a mesenchymal phenotype is not currently considered when stratifying recurrence risk or deciding on application of adjuvant chemotherapy.

Transcription factors that belong to the zinc finger E box–binding homeobox (ZEB) families are potent inducers of EMT^7^ and comprise 2 genes, *ZEB1* (OMIM 189909) and *ZEB2* (OMIM 605802).^18^ Aberrant expression of these genes is associated with poor outcomes in a number of malignant tumors.^9,11,19^ To date, their potential contribution as a biomarker to improve the ability of TNM staging in identifying patients at high recurrence risk after curative surgery for CRC has not been investigated. This study investigates the association of the EMT-inducing transcription factor ZEB2, survival outcomes, and the efficacy of ZEB2 as a biomarker when added as refinement to TNM staging after curative intent surgery for CRC.

## Methods

### Setting and Participants

All patients were recruited as part of an ongoing, prospective UK National Institute of Health Research Clinical Research Network study that is investigating the molecular pathology of CRC and was designed to identify novel biomarkers. Details from this ongoing study have been described previously.^20,21,22,23^ Study oversight and monitoring were performed at an independent clinical research organization. All patients provided written informed consent. All data were deidentified. After recruitment and surgery, tissue samples were deposited in a UK Human Tissue Act–approved tumor bank. Pathologic verification of diagnosis and staging was applied in accordance with the Association of Coloproctology of Great Britain and Ireland guidelines.^24^ The South Central Ethics Committee (Bristol, United Kingdom) approved this study.

In this study, all patients were recruited from January 1, 2008, to December 31, 2013, after referral to a regional academic university medical center, and data were prospectively collected and analyzed from January 1, 2017, to December 31, 2018. Paraffin-embedded tissue specimens were retrieved for all patients in the current study. Exclusion criteria were evidence of a hereditary tumor, R1/R2 surgical resection, presence of multiple tumors, tumors with histologically identified extensive necrosis, and tumors with synchronous metastases at presentation. The database was queried for information that related to patient demographics, preoperative risk, imaging, surgery, pathologic features, postoperative management, and oncologic outcomes.

### Antibody Generation

The ZEB2 antibody used in this study has been described^9^ and validated previously^13,25^ and was used at a 1:750 concentration. The specificity of the antibody and applicability of the scoring criterion were previously validated and reported.^25^

### Immunohistochemical Analysis and Scoring Method

All immunohistochemical analyses were conducted at the histochemistry research unit at University Hospital Southampton, United Kingdom, using an automated immunostaining device (Autostainer XL, Leica Biosystems). Because ZEB2 is a nuclear transcription factor, all stained sections were assessed for the presence of nuclear ZEB2 expression in neoplastic and normal tissue. Mesenchymal cells, such as fibroblasts or lymphocytes, in the tissue naturally express ZEB2 and served as a positive control.^9,13,25^ Two independent, masked pathologists (G.T. and K.M.) scored the sections as ZEB2 positive or negative using previously established scoring criteria.^25^ When a disparity in scores was noted, slides were rereviewed to reach a consensus. ZEB2 expression was correlated with oncologic outcomes to evaluate its role as a biomarker. All results have been reported in line with reporting standards for biomarker development proposed by the Reporting Recommendations for Tumor Marker Prognostic Studies (REMARK) and the Transparent Reporting of a Multivariable Prediction Model for Individual Prognosis or Diagnosis (TRIPOD) reporting guideline (eTables 1 and 2 in the Supplement).^26^

### Survival Analyses, Power Calculation, and Open Access Gene Expression Analysis

SPSS statistical software, version 22 (IBM Corp) was used to undertake survival analysis and examine the association between clinical and pathologic features and ZEB2 expression. Pathologists masked to patient outcome performed all biomarker scoring. Primary study end points of overall survival (OS) and disease-free survival (DFS) were defined as the time from date of primary resection to the date of death for OS or recurrence for DFS. Patient outcomes are represented as Kaplan-Meier survival curves and differences in survival outcomes calculated using the log-rank test. Multivariable analysis using a Cox proportional hazards regression model was used to investigate the prognostic value of ZEB2 in a model that encompassed conventional pathologic risk factors, and hazard ratio (HR) tables were used to compare differences. The association of ZEB2 with clinical and pathologic factors was determined using a χ^2^ or Fisher exact test as appropriate. Power calculation was performed using nQuery statistical software (Statistical Solutions Ltd). The test cohort for ZEB2 biomarker evaluation consisted of 126 consecutive patients. On the basis of the test cohort, a power calculation identified a minimum sample size of 180 as a requirement to achieve 80% power using a 2-sided test and a significance of 5%, assuming an HR of 2.0. The final figure recruited for the validation cohort was 210 consecutive patients. Additional details of settings and participants are reported in eTable 3 in the Supplement. The reported *P* values are 2-sided, and statistical significance was set to *P* < .05 for all tests in the study. The HRs are presented with 95% CIs. External validity was evaluated using the open access gene expression portal PROGgeneV2,^27^ which is compiled from Gene Expression Omnibus, the European Bioinformatics Institute’s ArrayExpress, and The Cancer Genome Atlas. Messenger RNA (mRNA) expression profiles were queried for ZEB2 and disease relapse. Kaplan-Meier survival curves were generated and statistical significance was calculated by the log-rank test.

### Mathematical Modeling and Nomogram Generation

A binary logistic regression model composed of conventional pathologic risk factors with or without the addition of the ZEB2 score was used to construct nomograms in the test cohort. The validity of the model was investigated by applying the model to the validation cohort. The capacity to determine the risk of distant recurrence within 3 years of surgical resection was calculated before and after the addition of ZEB2 to TNM staging criterion, differentiation, and extramural vascular invasion. The capacity of ZEB2 to determine outcome when added to the nomograms is reported as a concordance index (C index), and the contribution of adding ZEB2 to the nomograms is reported as incremental area under the curve (iAUC) in accordance with American Joint Committee on Cancer guidelines.^6^

The C index estimates the probability of concordance between the estimated and observed outcomes in rank order and equivalent to the area under the receiver operating characteristic curve. The C index represents the ability of the model to discriminate between patients who developed distant recurrence and those who did not. The iAUC represents the improvement in the C index as a result of addition of the ZEB2 expression score. With an equal interest in sensitivity and specificity, the optimum thresholds were selected to generate 2 risk scores with and without ZEB2 expression status. Patients scoring equal to or above the threshold were classified as high risk and below the threshold as low risk. Kaplan-Meier and calibration plots were then generated to assess the clinical utility of the risk score. An identical method was followed during subset analysis of patients with node-negative disease. Risk factors used in the stages I to II model included the following: age, American Society of Anesthesiologists class, presence of bowel obstruction, presence of bowel perforation, T stage, differentiation, extramural vascular invasion, perineural invasion, and lymphatic invasion with or without ZEB2 score. The aim of constructing these nomograms was to investigate whether the addition of ZEB2, a mesenchymal cancer cell marker, increases the ability to identify patients at higher risk of early recurrence when used in conjunction with conventional pathologic or clinical risk factors.

## Results

### Patient Demographics

The test cohort consisted of 126 consecutive patients (mean [SD] age, 72.7 [11.7] years; 61 [48.4%] male) and the validation cohort of 210 patients (mean [SD] age, 72.0 [10.6] years; 111 [52.9%] male). Patient demographics for the cohorts are provided in eTable 3 in the Supplement. In the test cohort, 89 patients (70.6%) had colonic carcinomas and 33 (26.2%) had rectal carcinomas. Disease staging was 1 or 2 in 77 patients, and 48 had lymph node metastasis (median lymph node sample of 14). In the validation cohort, 159 patients (75.7%) had colonic carcinomas and 51 (24.3%) had rectal carcinomas (eTable 3 in the Supplement). A total of 67 patients (31.9%) had lymph node metastasis (median node harvest of 15). A total of 92% of all rectal cancers were resected in the mesorectal plane.

### Association of Nuclear ZEB2 With Early Recurrence and Reduced Survival in the Test Cohort

**Figure 1.**
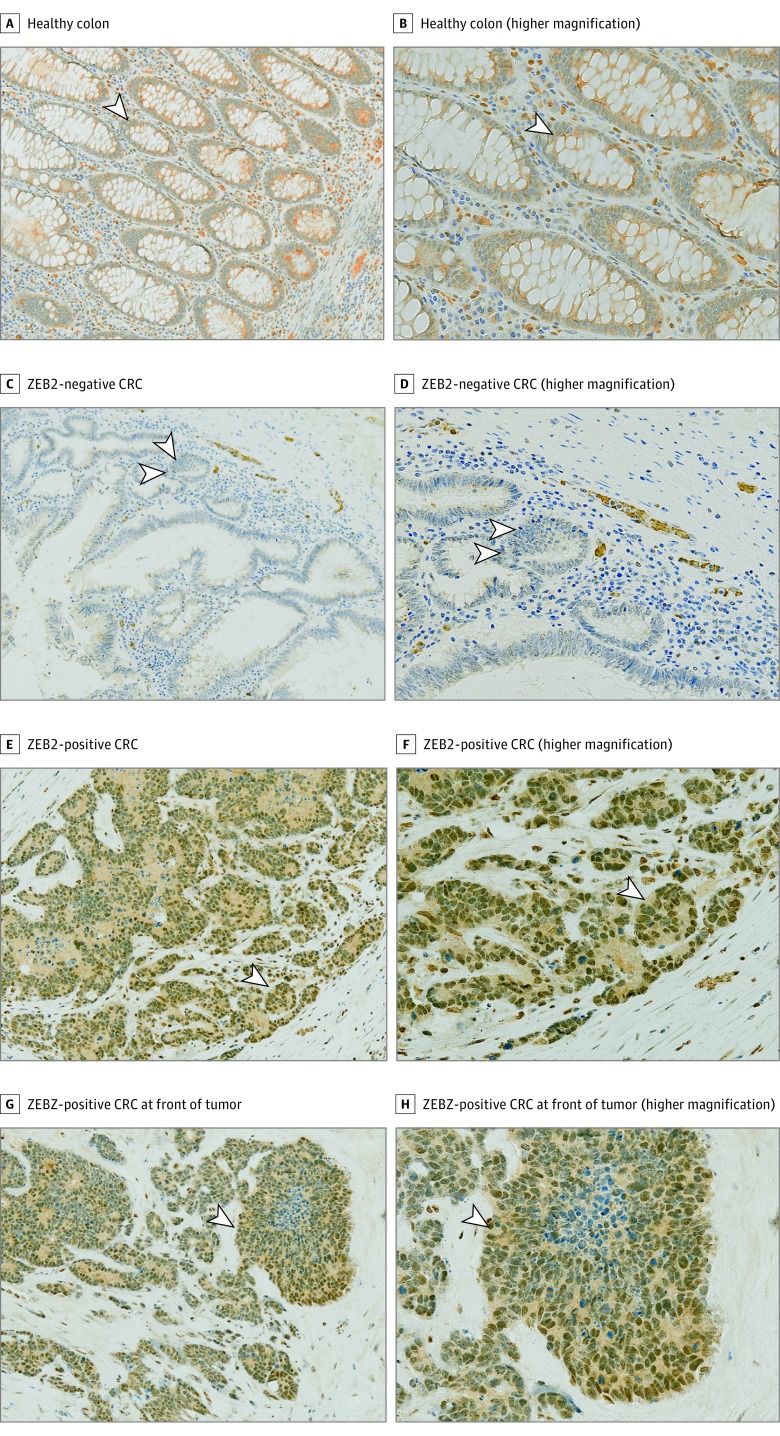
Immunohistochemical Analysis of ZEB2 Expression in Healthy Colon and Colorectal Cancer (CRC) Specimens A and B, Healthy colonic epithelium. The arrowheads indicate cells with an absence of ZEB2 nuclear staining (A) and fibroblasts and immune cells with positive nuclear staining that served as internal positive controls (B). C and D, Representative example of a CRC specimen that stained negative for ZEB2. Arrowheads indicate cells with an absence of ZEB2 nuclear staining (C) and fibroblasts and immune cells that served as internal positive controls (D). E and F, Representative example of a CRC specimen that stained positive for ZEB2. Arrowheads indicate CRC specimen expressing nuclear ZEB2 in neoplastic cells (E) and staining of cells in the middle of the cluster of neoplastic cells and evidence of the specificity of the antibody (F). G and H, A CRC specimen that was ZEB2 positive at the invasive front of the tumor. Immunohistochemical analysis for ZEB2 (brown) was performed on all images, visualized by 3,3′-diaminobenzidine–positive chromogen, and counterstained with hematoxylin (blue) (original magnification ×100 [A, C, E, and G] and ×200 [B, D, F, and H]). Arrowheads indicate nuclear positivity.

Immunohistochemical analysis was performed on the test cohort and ZEB2 expression and positivity recorded using a previously described scoring system.^9^ The REMARK and TRIPOD biomarker standards for the present study are provided in eTables 1 and 2 in the Supplement. Representative images of ZEB2 immunostaining are shown in Figure 1. No ubiquitous expression of nuclear ZEB2 was detected in normal colonic epithelium (Figure 1A and B). Mesenchymal cells, such as fibroblasts or lymphocytes, naturally express ZEB2 and served as the positive control. Two pathologists (G.T. and K.M.) masked to groups and outcomes scored 52 of the 126 specimens (41.3%) as ZEB2 positive (Figure 1C-H and eTable 3 in the Supplement).

**Figure 2.**
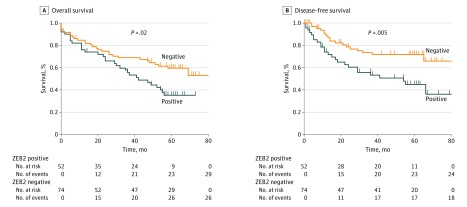
Association of ZEB2 With Early Recurrence and Reduced Survival in the Test Cohort Kaplan-Meier survival curves for the test cohort demonstrating differences in overall survival (A) and disease-free survival (B) when patients were stratified as ZEB2 negative or positive. *P* values were calculated using the log-rank test.

Clinical and pathologic evaluation revealed a potential association between ZEB2 positivity and lymph node metastasis and higher-stage disease (eTable 4 in the Supplement). No association was observed between ZEB2 expression and differentiation, age, sex, or T stage. Survival analyses by log-rank test highlighted increased recurrence (Figure 2B) and reduced survival (Figure 2A) in ZEB2-expressing patients. Mean OS among ZEB2-expressing patients was 43.8 months (95% CI, 34.4-49.7 months) compared with 60.4 months (95% CI, 50.0-65.8 months) among ZEB2-negative patients (log-rank test, *P* = .02). Mean DFS among ZEB2-positive patients was 48.0 months (95% CI, 38.2-54.0 months) compared with 60.5 months (95% CI, 56.6-72.5 months) among ZEB2-negative patients (log-rank test, *P* = .005). Multivariable analysis by Cox proportional hazards regression analysis highlighted ZEB2 as an independent biomarker of both OS (HR, 1.7; 95% CI, 1.1-2.8; *P* = .05) and DFS (HR, 2.2; 95% CI, 1.2-4.2; *P* = .05) (eTable 5A in the Supplement). Compared with ZEB2-negative patients, ZEB2-positive patients had a 1.7-fold increased risk of mortality and a more than 2-fold increased recurrence within 5 years of surgery.

### ZEB2 Analysis in Validation Cohort

**Figure 3.**
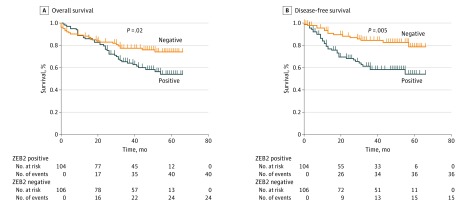
Association of ZEB2 With Early Recurrence and Reduced Survival in the Validation Cohort Kaplan-Meier survival curves for validation cohort demonstrating differences in overall survival (A) and disease-free survival (B) when patients were stratified as ZEB2 negative or positive. *P* values were calculated using the log-rank test.

Informed by the test cohort, a power calculation identified the need for a minimum of 180 patients in a validation cohort. The final validation cohort consisted of 210 consecutive patients to allow for exclusions. Patients with metastatic disease at diagnosis (n = 26) were excluded. Assessment of ZEB2 immunoexpression revealed 104 of 210 tumors (49.5%) to be ZEB2 positive (eTable 3 in the Supplement). Clinical and pathologic evaluation mirrored associations observed in the test cohort (eTable 4 in the Supplement). Survival analysis by log-rank testing maintained consistency with results from the test cohort. A mean 6.9-month reduction in OS (ZEB2-negative tumors vs ZEB2-positive tumors: 47.0 [95% CI, 42.3-51.7] vs 53.9 [95% CI, 48.8-57.9] months; log-rank test, *P* = .02) (Figure 3A) and a mean 13-month reduction (ZEB2-negative tumors vs ZEB2-positive tumors: 44.6 [95% CI, 38.9-50.0] vs 57.6 [95% CI, 53.1-61.3] months; log-rank test, *P* = .001) (Figure 3B) in DFS were observed among ZEB2-positive compared with ZEB2-negative patients. Multivariable analysis verified ZEB2 as an independent biomarker of OS (HR, 1.4; 95% CI, 1.2-2.1; *P* = .05) and DFS (HR, 3.0; 95% CI, 1.6-6.1; *P* = .001) (eTable 5B in the Supplement). T stage, N stage, and ZEB2 positivity were identified as independent biomarkers of OS, whereas T stage, N-stage differentiation, and ZEB2 status were independent biomarkers of DFS. We next investigated external validity of our findings using the open access gene expression portal PROGgeneV2, which is compiled from Gene Expression Omnibus, the European Bioinformatics Institute’s ArrayExpress, and The Cancer Genome Atlas. This search confirmed findings similar to our test and validation cohorts, with high ZEB2 expression associated with a greater risk of recurrence (HR, 2.1; 95% CI, 1.16-3.79; *P* = .02) (eFigure 1 in the Supplement).

### Association of Nuclear ZEB2 With Risk of Distant but Not Local Recurrence

Several in vitro and in vivo studies^9,28,29^ have reported that EMT results in an enhanced capacity to metastasize to distant organs. Accordingly, we evaluated study patients separately for distant recurrence as opposed to local recurrence and examined the association of ZEB2 in these contexts. Detailed definitions of distant recurrence and local recurrence are described in this study’s REMARK profile (eTable 1 in the Supplement).

Survival analysis in the test and validation cohorts showed that ZEB2 positivity selectively determines distant recurrence but not local recurrence (eFigures 2 and 3 in the Supplement), consistent with enhanced motility and migration characteristics associated with EMT. Mean time to distant recurrence was significantly shorter in ZEB2-positive patients in both cohorts. An 18-month reduction in mean time to distant recurrence (49.6 [95% CI, 40.1-59.1] vs 67.6 [95% CI, 59.9-75.2] months; log-rank test, *P* = .01) in the test cohort and a 11.4-month reduction (48.0 [95% CI, 42.6-53.5] vs 59.4 [55.6-63.1] months; log-rank test, *P = *.01) in the validation cohort were observed. In contrast, no difference in local recurrence was observed. Multivariable analysis identified ZEB2 as an independent biomarker of distant recurrence in the test (HR, 1.8; 95% CI, 1.4-3.7; *P* = .05) and validation (HR, 3.2; 95% CI, 1.6-6.6; *P* = .001) cohorts.

### Identification of Stages I to II Disease in Patients at High Risk of Recurrence

**Figure 4.**
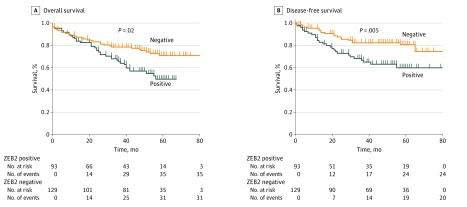
Association of ZEB2 With Early Recurrence and Reduced Survival in Stage I/II Disease Kaplan-Meier survival curves from analysis of patients with node-negative stage I/II disease, demonstrating differences in overall survival (A) and disease-free survival (B) when patients were stratified as ZEB2 negative or positive. *P* values were calculated using the log-rank test.

The benefit of administering adjuvant chemotherapy in stage III CRC is well recognized.^30^ Selecting node-negative patients (stages I to II disease) who benefit from adjuvant chemotherapy using conventional assessment remains suboptimal, however.^31^ Consequently, we next investigated whether ZEB2 expression in node-negative patients could identify patients at higher risk for recurrence. In total, 222 patients from the total study cohort were identified and cumulatively analyzed (demographics and clinicopathologic associations given in eTables 6 and 7 in the Supplement). ZEB2 expression in node-negative patients was associated with a significant reduction in OS and DFS. ZEB2-positive, node-negative tumors had a 19.4-month reduction in time to recurrence (mean, 55.5 [95% CI, 48.4-62.6] vs 74.9 [95% CI, 68.6-81.0] months; log-rank test, *P* = .001) and 23.8-month decrease in OS (mean, 46.4 [95% CI, 41.0-51.5] vs 70.2 [95% CI, 64.2-76.3] months; log-rank test, *P* = .008) (Figure 4). ZEB2 expression was associated with earlier distant recurrence (log-rank test, *P* = .01) but not local recurrence (log-rank test, *P* = .12).

An 18.2-month reduction (mean, 59.9 [95% CI, 53.1-66.8] vs 78.1 [95% CI, 72.3-83.9] months) in time to distant recurrence was observed in ZEB2-positive cases. Multivariable analysis incorporating known clinical and pathologic risk factors of stage I and II disease identified ZEB2 expression as an independent biomarker of distant recurrence (HR, 1.9; 95% CI, 1.6-6.6; *P* = .001) and OS (HR, 1.9; 95% CI, 1.2-3.2; *P* = .009) (eTable 8 in the Supplement). At 5 years, ZEB2-expressing patients were almost twice as likely to have experienced recurrence or death compared with ZEB2-negative patients. Further subgroup analyses to evaluate rectal and colon cancer separately found similar findings, and multivariable analysis identified ZEB2 as an independent biomarker (eFigure 4 in the Supplement).

### Addition of ZEB2 Expression to TNM Staging 

Our findings confirm and validate an association between ZEB2 expression and increased incidence of distant recurrence, independent of stage. To investigate whether the addition of ZEB2 expression to conventional TNM risk factors is associated with an improvement in the ability to identify patients at higher risk of early recurrence (<3 years) after curative surgery, nomograms and mathematical modeling with or without ZEB2 expression scores were developed.

Nomograms and modeling were developed in the test cohort and applied to our validation set. In the test cohort, the C index to identify distant recurrence within 3 years of surgery using conventional histologic risk factors was 0.73 (95% CI, 0.62-0.84) and improved to 0.77 (95% CI, 0.66-0.87; iAUC, 0.04) with the addition of ZEB2 expression.

**Figure 5.**
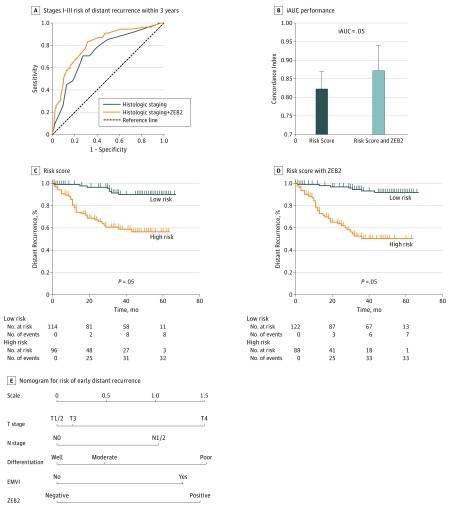
Improvement in the Ability to Stratify Patients for Risk of Recurrence After Curative Surgery With the Addition of ZEB2 Expression Status to the TNM Staging System A and B, Receiver operating characteristic curves were generated with and without addition of ZEB2 to the TNM staging system to evaluate any improvement in incremental area under the curve (iAUC). B, Improvement in iAUC secondary to the addition of ZEB2 as a histogram. Error bars indicate 95% CI. C and D, Kaplan-Meier curves show increased separation and stratification when ZEB2 is added to the nomogram. Patients were stratified as high risk or low risk based on scores generated from the nomogram. C, Results of the risk score alone. D, Results with the addition of ZEB2 scoring. *P* values were calculated using the log-rank test. E, Visual representation of the nomogram used to estimate risk of early distant recurrence. EMVI indicates extramural vascular invasion.

Application of the nomogram to the validation cohort (n = 210) showed excellent performance and ability to identify risk, highlighting an identical trend, with the C index improving from 0.82 (95% CI, 0.75-0.87) to 0.87 (95% CI, 0.80-94; iAUC, 0.05) (Figure 5A and B). Kaplan-Meier survival plots generated by applying the risk score (high risk, ≥1.4; low risk, <1.4) to the validation cohort revealed significantly greater separation (Figure 5C and D). Application of ZEB2 scoring resulted in improvement in sensitivity of 5.2% and specificity of 8.7%. These improvements would result in improved risk stratification in 14 of 100 patients assessed compared with use of the TNM staging system without inclusion of ZEB2-expression status. Calibration plots demonstrated good concordance between expected and observed outcome, and results of the Hosmer-Lemeshow test, used to detect differences in expected and observed events, were statistically insignificant (*P* = .84). Taken together, these observations suggest that ZEB2 expression, if used in conjunction with conventional TNM-based histologic staging, is associated with an improvement in the ability to identify patients at increased risk of experiencing distant recurrence after surgical resection.

The inability to stratify risk in patients with node-negative CRC impedes the ability to identify patients who will derive maximum benefit from adjuvant chemotherapy. We next investigated whether the addition of ZEB2 expression to existing high-risk factors used to determine the need for adjuvant chemotherapy in patients with node-negative CRC would further aid detection of patients at increased risk for recurrence. A multivariable logistic regression model that contained conventional pathologic and clinical risks was constructed to identify independent variables. Clinical and pathologic variables considered and incorporated into the model are listed in eTable 8 in the Supplement. In node-negative disease, the C indices were 0.83 (95% CI, 0.70-0.95) for estimation of distant recurrence and 0.84 (95% CI, 0.78-0.90) for estimation of OS at 3 years. The C index improved from 0.80 to 0.83 with the addition of ZEB2, with improvements in sensitivity of 3% and specificity of 7%. Consequently, application of ZEB2 scoring would improve risk stratification in 10 of every 100 node-negative patients.

## Discussion

Currently, CRC is treated as a genetically homogeneous disease. There are no molecular biomarkers in routine clinical use to stratify risk of recurrence after surgical excision of the primary tumor, and only *kras* mutational status is approved as a negative determinant in patients treated with epidermal growth factor receptor inhibitors.^32,33^ We demonstrate that ZEB2, an EMT-inducing transcription factor expressed in mesenchymal cancer cells, is associated with an increased risk of distant recurrence and reduced OS after curative surgery. Subset analysis in node-negative disease also highlighted ZEB2 as an independent biomarker. We next developed and internally validated, to our knowledge, the first nomogram for distant recurrence that incorporates this biomarker and found good estimation ability and superiority over conventional and currently used TNM risk factors.

Epithelial to mesenchymal transition is a critical event in cancer metastasis.^6,34^ The association among EMT, downregulation of epithelial adhesion molecules,^7^ modulation of the microenvironment to promote invasion,^35^ and cytoskeletal alterations that increase motility^7^ has long been known. Tissue-specific knockdown of critical EMT-inducing transcription factors (eg, ZEB1 in pancreatic cancer) eliminates the ability of cancer cells to metastasize.^28^ Furthermore, large-scale consortia of leading scientists have highlighted the importance of identifying tumors that express a mesenchymal phenotype.^5,14,15,16,17^ Consequently, biomarkers that detect an EMT phenotype in the primary tumor may help identify those at risk of recurrence attributable to harboring occult micrometastases. E-cadherin downregulation is a cardinal feature of EMT and has been extensively investigated as such a biomarker.^12,36,37^ However, studies^38,39^ that use E-cadherin have reported conflicting results.

Of the EMT transcription factors, ZEB proteins have been sparsely studied in CRC.^40^ Furthermore, to date, no prospective or validated biomarker studies have been undertaken, making clinical translation challenging. Kahlert et al^11^ previously reported cytoplasmic expression of ZEB2 at the invasive front of primary CRCs identified as having poor cancer-specific survival. However, nuclear expression, the ability to differentiate local recurrence from distant recurrence, or applicability in node-negative disease and ability to identify patients at high risk of distant recurrence were not considered. Our validated results emphasize the importance of nuclear positivity because ZEB proteins modulate gene expression through epigenetic regulation in the nucleus.^41^

A major advantage of ZEB2 scoring is its nondependence on specialized molecular testing. Immunohistochemical analysis is routinely performed in all clinical pathology laboratories and readily translated into clinical practice. Most gene expression platforms quantify mRNA expression and transcriptional changes. However, an in vitro study^42^ found that EMT is highly regulated by microRNAs; consequently, mRNA expression does not automatically result in protein expression and activation of EMT. A further limitation of polymerase chain reaction–based assays relates to tumor sampling; EMT often occurs in subsets of cancer cells within a tumor.^43^ Therefore, attempts to quantify aberrant expression by sampling a small area may fail to represent true genetic heterogeneity of any sample. This limitation can be reduced by analysis of multiple sections of a single tumor by immunohistochemical analysis.

A key additional recognized feature of EMT is the acquisition of chemoresistance to compounds routinely used in clinical practice.^9,44^ Therefore, it is possible that detection of a mesenchymal profile by ZEB2 expression not only identifies recurrence risk but also potential response to chemotherapeutic agents.

### Limitations

Although this study has demonstrated a consistent association among ZEB2 expression, early recurrence, and reduced survival in 3 independent cohorts, a few limitations need to be considered. Microsatellite stability, *kras*, and *BRAF* mutation status were not analyzed, and consequently, the association with ZEB2 was not studied. ZEB2 staining in the present study was based on surgical resection samples and not pretherapy biopsy samples. As a result, a small proportion of patients with CRC in both groups received neoadjuvant therapy, which may be a confounding factor. We did not note any association between ZEB2 status and neoadjuvant therapy and observed that even in the CRC cohort alone, ZEB2 was able to stratify patients; however, this study was not powered to address this subgroup formally. Finally, the association of the immune microenvironment with cancer outcomes is increasingly uncovered.^45,46^ Similarly, there is increasing evidence that mesenchymal cancer cells can modulate the immune infiltrate to promote tumor progression.^47^ Investigating the interdependence of immune scores and ZEB2 expression was beyond the scope of the current study; however, this may be critical in future efforts.

## Conclusions

We report, for the first time to our knowledge, that nuclear ZEB2 expression in CRC was associated with early recurrence and reduced survival in 2 independent prospective patient cohorts. ZEB2 identified patients at high risk of distant recurrence but not local recurrence, whereas subset analysis of node-negative disease revealed an identical association independent of stage. Addition of the ZEB2 score to nomograms composed of conventional TNM and histologic factors increased sensitivity and specificity of TNM, resulting in improved risk stratification in 14 of every 100 patients assessed.

Using independent testing, validation, and external cohorts and multivariable analysis, we found that ZEB2 expression in CRC may be associated with a high risk of distant recurrence after curative surgery. Detection of this molecular marker by immunohistochemical analysis is a simple and reproducible method for detecting a mesenchymal phenotype. ZEB2 expression was an independent biomarker in all cases, including patients with node-negative and rectal cancer. Using a novel nomogram, we found that the addition of ZEB2 expression status more accurately estimated prognosis than did TNM staging alone and that TNM staging with ZEB2 expression status may have utility in patient counseling, trial design, and clinical decision making. Additional external validation or incorporation in a randomized biomarker-driven study are needed to support modification of TNM staging by addition of this novel marker.

## Notes

Supplement. **eTable 1.** REMARK Biomarker Profile for the Present Study **eTable 2.** TRIPOD Checklist: Prediction Model Development **eTable 3.** Patient Demographics for Test and Validation Cohorts **eTable 4.** Association Between Clinico-Pathological Features and ZEB2 Expression **eTable 5.** Multi-variable Cox-regression analysis of overall and disease free survival in the test cohort (panel A) and validation cohorts (panel B), presented as hazard ratios (HR) with 95% confidence interval (CI). **eTable 6.** Patient Demographics for Stage I and II Disease **eTable 7.** Association Between Clinico-Pathological Features and ZEB2 Expression in Node-Negative Disease **eTable 8.** Multi-variable Cox-regression analysis of overall and disease free survival in the node-negative disease, presented as hazard ratios (HR) with 95% confidence interval (CI) **eFigure 1.** External validity was investigated using the open access portal PROGgeneV2, which is compiled from GEO, EBI array express, and the The Cancer Genome Atlas. **eFigure 2.** Nuclear ZEB2 associates with risk of distant (A) but not local (B) recurrence in test cohort. **eFigure 3.** Nuclear ZEB2 associates with risk of distant (A) but not local (B) recurrence in validation cohort. **eFigure 4.** ZEB2 is an Independent Prognostic Marker of Survival in Patients with Non-Metastatic Rectal Cancer (Sub-Group Analysis)
