# Supplementary material for: Assessment of Nuclear ZEB2 as a Biomarker for Colorectal Cancer Outcome and TNM Risk Stratification
Source: JAMA Netw Open. 2018 Oct 5;1(6):e183115. doi: 10.1001/jamanetworkopen.2018.3115 (PMC6324431; doi:10.1001/jamanetworkopen.2018.3115)
Supplement: Supplement. — eTable 1. REMARK Biomarker Profile for the Present Study eTable 2. TRIPOD Checklist: Prediction Model Development eTable 3. Patient Demographics for Test and Validation Cohorts eTable 4. Association Between Clinico-Pathological Features and ZEB2 Expression eTable 5. Multi-variable Cox-regression analysis of overall and disease free survival in the test cohort (panel A) and validation cohorts (panel B), presented as hazard ratios (HR) with 95% confidence interval (CI). eTable 6. Patient Demographics for Stage I and II Disease eTable 7. Association Between Clinico-Pathological Features and ZEB2 Expression in Node-Negative Disease eTable 8. Multi-variable Cox-regression analysis of overall and disease free survival in the node-negative disease, presented as hazard ratios (HR) with 95% confidence interval (CI) eFigure 1. External validity was investigated using the open access portal PROGgeneV2, which is compiled from GEO, EBI array express, and the The Cancer Genome Atlas. eFigure 2. Nuclear ZEB2 associates with risk of distant (A) but not local (B) recurrence in test cohort. eFigure 3. Nuclear ZEB2 associates with risk of distant (A) but not local (B) recurrence in validation cohort. eFigure 4. ZEB2 is an Independent Prognostic Marker of Survival in Patients with Non-Metastatic Rectal Cancer (Sub-Group Analysis) [file jamanetwopen-1-e183115-s001.pdf]

Supplementary Online Content

Sreekumar R, Harris S, Moutasim K, et al. Assessment of Nuclear ZEB2 as a Biomarker for Colorectal Cancer Outcome and TNM Risk Stratification. *JAMA Netw Open*. 2018;1(6):e183115. doi:10.1001/jamanetworkopen.2018.3115

eTable 1. REMARK Biomarker Profile for the Present Study

eTable 2. TRIPOD Checklist: Prediction Model Development

eTable 3. Patient Demographics for Test and Validation Cohorts

eTable 4. Association Between Clinico-Pathological Features and ZEB2 Expression

eTable 5. Multi-variable Cox-regression analysis of overall and disease free survival in the test cohort (panel A) and validation cohorts (panel B), presented as hazard ratios (HR) with 95% confidence interval (CI).

eTable 6. Patient Demographics for Stage I and II Disease

eTable 7. Association Between Clinico-Pathological Features and ZEB2 Expression in Node-Negative Disease

eTable 8. Multi-variable Cox-regression analysis of overall and disease free survival in the node-negative disease, presented as hazard ratios (HR) with 95% confidence interval (CI)

eFigure 1. External validity was investigated using the open access portal PROGgeneV2, which is compiled from GEO, EBI array express, and the The Cancer Genome Atlas.

eFigure 2. Nuclear ZEB2 associates with risk of distant (A) but not local (B) recurrence in test cohort.

eFigure 3. Nuclear ZEB2 associates with risk of distant (A) but not local (B) recurrence in validation cohort.

eFigure 4. ZEB2 is an Independent Prognostic Marker of Survival in Patients with Non-Metastatic Rectal Cancer (Sub-Group Analysis)

This supplementary material has been provided by the authors to give readers additional information about their work.

© 2018 Sreekumar R et al. *JAMA Network Open.*

eTable 1

REMARK Biomarker Profile for the Present Study

**REMARK guidelines for biomarker reporting**	
Biomarker examined	ZEB2
Disease studied	Colorectal adenocarcinoma
Database	Prospective
Time period	2007-2013
Hypothesis	Nuclear ZEB2 expression predicts early distant recurrence and reduced survival independent of stage in colorectal cancer.
Inclusion criteria	Non Metastatic colorectal adenocarcinoma + Curative Surgical resection
Exclusion criteria	Synchronous metastasis / R1/R2 resection, Multiple tumour/ Hereditary disease
Treatment	Surgical resection +/- Adjuvant therapy
Biological material	Paraffin embedded human tissue
Biomarker detection	Automated Immunohistochemistry / Leica XL Auto-stainer
Antibody	In-house/Rabbit/Polyclonal/1:750 dilution
Quality control	Antibody optimisation on uterine myometrium<br>Positive Control – Fibroblasts / Uterine tissue<br>Negative control – Normal colon
Scoring	2 independent blinded pathologists / Nuclear ZEB2 staining / >10% of cancer cells / Positive or Negative
Median follow up<br>Training Cohort<br>Validation Cohort	 50 months<br>45 months
Clinical end points and definitions	Overall survival – time to death (Clinical records) from date of surgery<br>Disease free survival – Time to detection of recurrence from date of surgery<br>Distant recurrence – Radiological detection of metastasis outside the colon after surgery<br>Local recurrence – Radiological evidence of recurrence at the site of surgical resection<br>Locally recurrent rectal cancer – defined according to the Beyond TME collaborative definition[29]
Subgroup analysis	Node negative disease
Cox regression model	Multiple, Refer to main text

© 2018 Sreekumar R et al. *JAMA Network Open.*

eTable 2

TRIPOD Checklist: Prediction Model Development

Section/Topic		Checklist Item	Page
**Title and abstract**			
Title	1	Assessment of Nuclear ZEB2 as a biomarker for colorectal cancer outcome and TNM risk stratification	
Abstract	2	**Objectives:** The objective of this study was to investigate the association between expression of the epithelial to mesenchymal transition (EMT) inducing transcription factor ZEB2, survival outcomes and its efficacy as a biomarker when added as refinement to TNM staging, after curative intent surgery for CRC.<br>**Study design:** Prospective observational cohort study<br>**Setting:** Academic University medical centre<br>**Participants:** Stage I-III colorectal adenocarcinoma + curative surgical resection.<br>**Sample size:** Test Cohort (n=126), Validation Cohort (n=210), External validity tested using the open access gene portal. PROGgeneV2.<br>**Predictors:** ZEB2<br>**Outcome:** Overall survival (OS), disease free survival (DFS), Local recurrence (LR) and Distant Recurrence (DR)<br>**Statistical analysis:** Univariate (Kaplan Meier, Log rank), Multi-variable (Cox-regression), Nomogram (Binary logistic regression, ROC cuve)<br>**Results:** ZEB2 associates with poor oncological outcomes and improves ability to stratify patients for risk of recurrence	
**Introduction**			
Background and objectives	3a	Currently patients are risk stratified using TNM staging system. Emerging evidence suggests expression of a mesenchymal phenotype associates with poor oncological outcome in patients with colorectal adenocarcinoma. EMT inducing transcription factor (TF) ZEB2 has been reported to associate with poor oncological outcomes in multiples cancers. This study investigated the association of ZEB2 with survival outcomes in CRC and analyses its contribution towards optimising risk stratification when added to the TNM staging system.	
	3b	The objective of this study was to investigate the association between expressions of the epithelial to mesenchymal transition (EMT) inducing transcription factor ZEB2, survival outcomes and its contribution towards refinement of TNM staging. The study outlines both development and validation of ZEB2 as a biomarker that refines stratification of disease risk when incorporated into TNM staging system.	
**Methods**			
Source of data	4a	Study design: Prospective observational cohort study<br>Source of data: Patients that underwent curative surgical resection for CRC at an academic university medical centre. Test Cohort (126), Validation cohort (n=210).	
	4b	Data accrual: 2008-2013<br>Data analysis: 2017-2018	
Participants	5a	Setting: Single academic university centre in the United Kingdom	
	5b	Inclusion: Stage I-III Colorectal adenocarcinoma + Curative resection<br>Exclusion: Synchronous metastasis / R1/R2 resection, Multiple tumour/ Hereditary disease	
	5c	Curative surgical resection +/- Neo-adjuvant / Adjuvant Chemo/radiotherapy	
Outcome	6a	Distant Recurrence	
	6b	N/A	
Predictors	7a	TNM +/- ZEB2	
	7b	N/A	
Sample size	8	Based on the test cohort, a power calculation identified a minimum sample size of 180 as a requirement to achieve 80% power using a two-sided test and a significance of 5%, assuming a hazard ratio of 2.0.	
Missing data	9	Complete-case analysis, No imputation methods used	
Statistical analysis methods	10a	All predictors were categorised as represented in the Nomogram in Figure 5	
	10b	TNM was used as the reference model as it represents current standard of clinical practice. Model was developed using binary logistic regression method, TNM and TNM+ZEB2 models were developed using the test cohort and validity investigated by applying the model to the validation cohort.	
	10d	Model performance was compared using Concordance index (C-index) and incremental area under the curve (iAUC).	
Risk groups	11	With an equal interest in sensitivity and specificity, the optimum thresholds were selected to generate two risk scores with and without ZEB2 expression status. Patients scoring equal to or above the threshold were classified as high risk and below the threshold low risk.	
**Results**			
Participants	13a	Represented as Kaplan Meier survival curves with No. at risk tables in Figure 5	
	13b	Supplementary eTable 2 table	
	14a	Represented in Figure 5 B/C no. at risk tables	

© 2018 Sreekumar R et al. *JAMA Network Open.*

Model development	14b	Not Performed	
Model specification	15a	Represented In Nomogram Figure 5E	
	15b	Patients are divided into Low or high risk. Patients with a score equal to or above the threshold core (1.4) are categorised as high risk. The Nomogram provides a visual representation of risk addition score added by each histological feature and ZEB2 expression.	
Model performance	16	C- Index in the Test Cohort improved from 0.73 (95% CI 0.62-0.84 to 0.77 (95% CI 0.66-0.87, iAUC=0.04) with addition of ZEB2 expression. In the Validation Cohort, the c-index improved from 0.82 (95% CI 0.75-0.87) to 0.87 (95% CI 0.80-94)iAUC-0.05	
**Discussion**			
Limitations	18	Single EMT inducing TF studied, MSI status not known for the samples.	
Interpretation	19b	ZEB2 expression associates with poor disease free survival, with specificity to distant recurrence and improves capacity to stratify patients at risk of recurrence when incorporated into the TNM staging system.	
Implications	20	Patients considered high risk, regardless of node involvement could be considered for adjuvant chemotherapy. Evolution of new chemotherapeutic agents that specifically target tumours with a mesenchymal phenotype could potentially be selectively administered to patients that express ZEB2 in future years.	
**Other information**			
Supplementary information	21	N/A	
Funding	22	Medical Research Council.	

© 2018 Sreekumar R et al. *JAMA Network Open.*

eTable 3: Patient Demographics for Test and Validation Cohorts

	Test cohort		Validation cohort	
	**n**	**%**	**n**	**%**
**Age (Yrs)**				
<60	11	8.7	25	11.9
>60<br>Missing	115<br>0	91.3<br>0	185<br>0	88.1<br>0
**Sex**				
Male	61	48.4	111	52.9
Female	65	51.6	99	47.1
**ASA grade**				
1	9	7.1	21	10.0
2	56	44.4	95	45.2
3	34	27.0	82	39.0
4<br>Missing	5<br>22	4.0<br>17.4	7<br>5	3.3<br>2.4
**Site of tumours**				
Right	49	38.9	96	45.7
Left	40	31.7	63	30.0
Rectum<br>Missing	33<br>4	26.2<br>3.2	51<br>0	24.3<br>0
**Differentiation**				
Well	2	1.6	25	11.9
Moderate-well	47	37.3	4	1.9
Moderate	54	42.9	103	49.0
Moderate-poor	8	6.3	72	34.3
Poor<br>Missing	14<br>1	11.1<br>0.8	4<br>2	1.9<br>1.0
**Stage**				
Stage 1	22	17.5	32	15.2
Stage 2	55	43.7	111	52.9
Stage 3	48	38.1	67	31.9
Stage 4<br>Missing	0<br>1	0<br>0.8	0<br>0	0<br>0
**T-stage**				
T1	10	7.9	6	2.9
T2	16	12.7	42	20
T3	30	23.8	108	51.4
T4<br>Missing	69<br>1	54.8<br>0.8	54<br>0	25.7<br>0
**N-Positivity**				
N0	78	61.9	143	68.1
N1/N2<br>Missing	48<br>1	38.1<br>0.8	67<br>0	31.5<br>0
**Adjuvant Chemotherapy**				
Yes	82	65.0	85	40.5
No	42	33.3	125	59.5
Missing	2	1.6	0	0
**ZEB2 Positive**				
Yes	52	41.3	104	49.5
No	74	51.7	106	50.5

© 2018 Sreekumar R et al. *JAMA Network Open.*

eTable 4

Association Between Clinico-Pathological Features and ZEB2 Expression

	Training Cohort (n=126)			Validation cohort (n=210)		
**Characteristic**	**ZEB2 positive**	**ZEB2 negative**	**p-value**	**ZEB2 positive**	**ZEB2 negative**	**p-value**
**Age**						
**<60**	3	8	p=0.52	15	11	p=0.52
**>60**	49	66		89	95	
**Sex**						
**M**	20	41	p=0.06	51	60	p=0.27
**F**	32	33		53	46	
**T-stage**						
**T1**	3	7	p=0.62	4	2	p=0.16
**T2**	5	11		14	28	
**T3**	12	18		56	52	
**T4**	32	37		30	24	
**Nodal Positivity**						
**NO**	26	51	p=0.06	61	43	p<0.05
**N1/N2**	26	22		75	31	
**Stage**						
**1**	6	16	p=0.06	11	21	p<0.05
**2**	20	35		52	59	
**3**	26	22		41	26	
**4**	0	0		0	0	
**Differentiation**						
**Well**	1	1	p=0.95	2	4	p=0.19
**Moderate-well**	18	29		49	54	
**Moderate**	23	31		34	38	
**Moderate-poor**	3	5		4	0	
**Poor**	7	7		15	10	

© 2018 Sreekumar R et al. *JAMA Network Open.*

eTable 5

Multi-variable Cox-regression analysis of overall and disease free survival in the test cohort (panel A) and validation cohorts (panel B), presented as hazard ratios (HR) with 95% confidence interval (CI).

A

Test cohort (n=126)						
**Characteristic**	**Overall survival (OS)**			**Disease free survival (DFS)**		
	**HR**	**95% CI**	**p-value**	**HR**	**95% CI**	**p-value**
**Age (<60 vs. >60)**	2.3	0.6 - 9.2	0.24	1.00	0.2 – 4.4	1.00
**T-stage (Overall)**			<0.05			<0.05
**T stage (T1/2 vs. T4)**	2.1	0.9 – 4.5	0.06	4.30	1.3 – 14.6	<0.05
**T stage (T3 vs. T4)**	1.1	0.3 – 2.0		0.95	0.2 – 4.8	0.96
**N-stage (N0 vs. N1/2)**	1.4	0.4 - 1.7	0.32	1.20	0.6 – 2.3	0.70
**Differentiation**			<0.01			0.52
**Differentiation (Well vs. Poor)**	3.4	1.7 – 6.8	<0.01	1.55	0.4 – 1.9	0.36
**Differentiation (Mod vs. Poor)**	1.0	0.6 – 1.9	0.89	0.91	0.6 – 4.0	0.81
**ZEB2 Status (Pos vs. Neg)**	1.7	1.1 - 2.8	<0.05	2.2	1.2–4.2	<0.05

B

Validation cohort (n=210)						
	**Overall survival (OS)**			**Disease free survival (DFS)**		
**Characteristic**	**HR**	**95% CI**	**p-value**	**HR**	**95% CI**	**p-value**
**Age (<60 vs. >60)**	1.4	0.6-3.4	0.45	1.2	0.5-3.7	0.72
**T-stage (Overall)**			<0.05			<0.01
**T stage (T1/2 vs. T4)**	2.8	1.2-6.3	<0.05	4.3	1.5-12.4	<0.01
**T stage (T3 vs. T4)**	1.5	0.7–3.1	0.32	1.3	0.5-3.5	0.65
**N-stage (N0 vs. N1/2)**	1.8	1.1-2.9	<0.05	3.1	1.6-6.6	<0.01
**Differentiation**			0.86			<0.01
**Differentiation (Well vs. Poor)**	1.2	0.6-2.5	0.64	4.3	2.3-13.2	<0.01
**Differentiation (Mod vs. Poor)**	1.1	0.5-2.4	0.89	3.3	0.8-5.4	0.07
**ZEB2 Status (pos vs. neg)**	1.4	1.2-2.1	<0.05	3.0	1.6-6.1	<0.05

© 2018 Sreekumar R et al. *JAMA Network Open.*

eFigure 1

External validity was investigated using the open access portal PROGgeneV2, which is compiled from GEO, EBI array express, and the The Cancer Genome Atlas. mRNA gene expression profile from the database GSE28814 (PMID 21251323) confirmed similar findings to our test and validation cohorts, with high ZEB2 expression associated with a greater risk of recurrence (HR=2.1, 95% CI 1.16-3.79, $p<0.02$). Prognostic importance is demonstrated by Kaplan Meier survival curves and statistical significance calculated by log rank test.

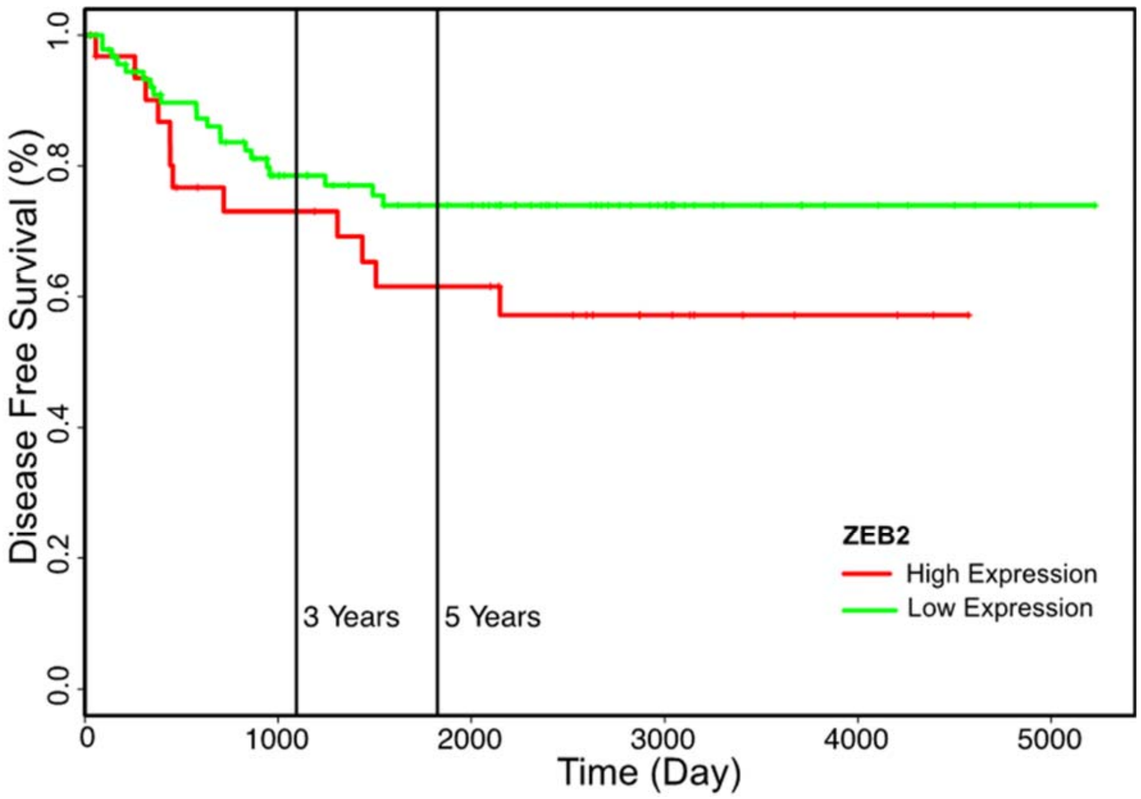

© 2018 Sreekumar R et al. *JAMA Network Open.*

eFigure 2

Nuclear ZEB2 associates with risk of distant (A) but not local (B) recurrence in test cohort. Kaplan-Meier curves were generated by differentiating a DFS event as either distant or local recurrence. ZEB2 expression associated with increased risk of distant but not local recurrence.

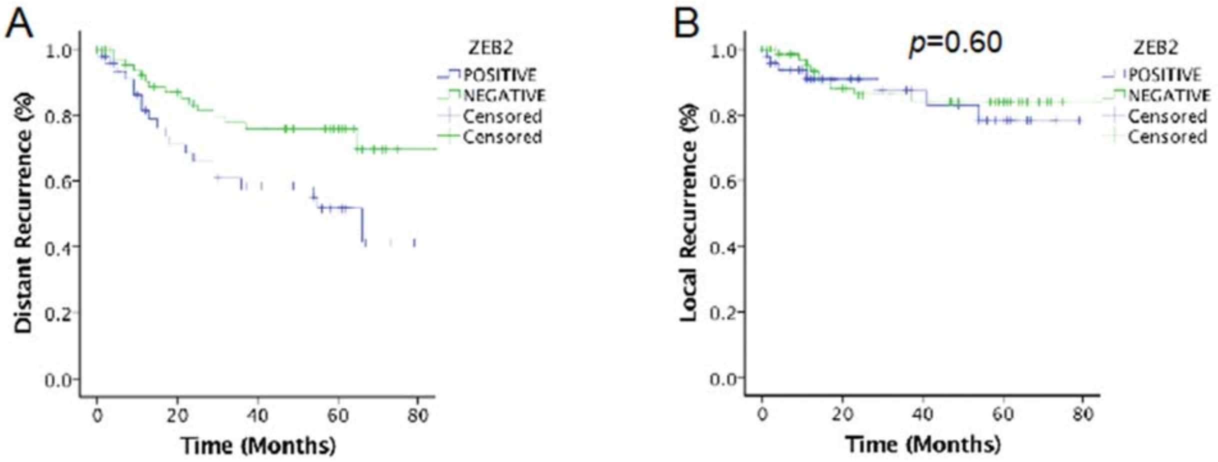

Months	0	20	40	60	80
ZEB2 +VE No. at risk	52	28	20	11	0
Events	0	12	17	19	20
ZEB2 –VE No. at risk	74	48	28	8	0
Events	0	8	14	15	15

Months	0	20	40	60	80
ZEB2 +VE No. at risk	52	28	19	10	0
Events	0	4	6	7	7
ZEB2 –VE No. at risk	74	47	41	29	0
Events	0	7	9	9	9

© 2018 Sreekumar R et al. *JAMA Network Open.*

eFigure 3

Nuclear ZEB2 associates with risk of distant (A) but not local (B) recurrence in validation cohort. Kaplan-Meier curves were generated by differentiating a DFS event as either distant or local recurrence. ZEB2 expression associated with increased risk of distant but not local recurrence.

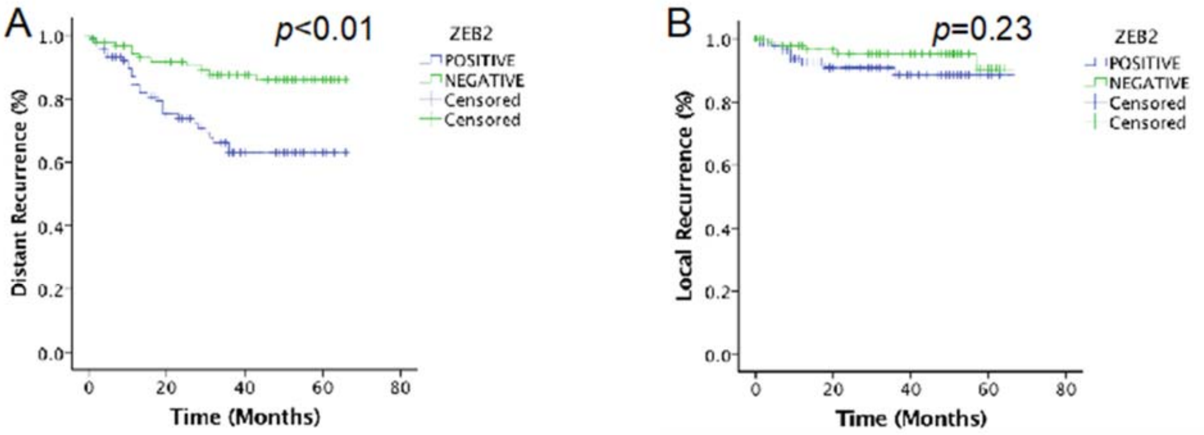

Months	0	20	40	60	80
ZEB2 +VE No. at risk	104	55	33	5	0
Events	0	20	28	28	28
ZEB2 –VE No. at risk	106	72	51	12	0
Events	0	7	10	11	11

Months	0	20	40	60	80
ZEB2 +VE No. at risk	104	55	33	6	0
Events	0	8	9	11	28
ZEB2 –VE No. at risk	106	72	52	12	0
Events	0	3	4	5	5

© 2018 Sreekumar R et al. *JAMA Network Open.*

eTable 6: Patient Demographics for Stage I and II Disease

Stage I&II patients		
**Characteristic**	**n**	**%**
**Age (Yrs)**		
<60	23	10.4
>60<br>Missing	199<br>0	89.6<br>0
**Sex**		
Male	116	53.3
Female<br>Missing	106<br>0	47.7<br>0
**ASA grade**		
1	17	7.6
2	87	39.1
3	94	42.3
4	7	3.1
Missing	17	7.7
**Site of tumours**		
Right	87	39.2
Left	69	31.0
Rectum	62	27.9
Missing	4	1.8
**Differentiation**		
Well	6	2.7
Moderate-well	107	48.2
Moderate	81	36.5
Moderate-poor	5	2.3
Poor	23	10.4
**T-stage**		
T1	12	5.4
T2	47	21.2
T3	99	44.6
T4	64	28.8
**Nodes Sampled**		
<12	95	42.8
>12	125	56.3
Missing	1	0.9
**Perineural/Lymphatic/EMVI**		
Positive	44	19.8
Negative	173	77.9
Missing	5	2.3
**Adjuvant Chemotherapy**		
Yes	54	24.8
No	167	74.8
Missing	1	0.4
**ZEB2 Positive**		
Yes	93	41.9
No	129	58.1

© 2018 Sreekumar R et al. *JAMA Network Open.*

eTable 7

Association Between Clinico-Pathological Features and ZEB2 Expression In Node-Negative Disease

Characteristic	ZEB2 positive	ZEB2 negative	p-value
**Age**			
<60	10	13	**p=0.870**
>60	83	116	
**Sex**			
M	45	71	**p=0.32**
F	48	58	
**T-stage**			
T1	3	9	**p=0.50**
T2	17	30	
T3	44	55	
T4	29	35	
**Nodes Sampled**			
<12	37	58	**p=0.45**
>12	55	70	
**Lymphatic / Perineural /EMVI**			
Yes	22	22	**p=0.23**
No	72	104	
**Differentiation**			
Well	19	9	**p<0.01**
Moderate	29	52	
Poor	45	68	

© 2018 Sreekumar R et al. *JAMA Network Open.*

eTable 8

Multi-variable Cox-regression analysis of overall and disease free survival in the node-negative disease, presented as hazard ratios (HR) with 95% confidence interval (CI). EMVI, Extramural vascular invasion.

	Overall survival (OS)			Disease free survival (DFS)		
**Characteristic**	**HR**	**95% CI**	**p-value**	**HR**	**95% CI**	**p-value**
**Age (<60 vs. >60)**	1.82	0.5–6.1	0.33	1.06	0.5–6.1	0.33
**T-stage (Overall)**			<0.01			<0.01
**T stage (T1/2 vs. T4)**	4.12	1.2–4.5	<0.01	4.00	1.2–4.5	<0.01
**T stage (T3 vs. T4)**	1.24	0.5–2.1	0.84	1.00	0.5 – 2.1	0.13
**Nodes sampled (<12 vs >12)**	1.52	0.9–2.5	0.09	1.19	0.9–2.5	0.09
**EMVI (positive vs. negative)**	2.10	1.1–2.4	<0.05	2.82	0.9–2.4	0.03
**Differentiation**			0.28			<0.01
**Differentiation (Well vs. Poor)**	0.82	0.3–1.9	0.64	7.64	1.5–7.8	<0.01
**Differentiation (Mod vs. Poor)**	1.27	0.5–2.4	0.54	2.83	0.5–4.8	0.22
**Chemotherapy (Yes vs No)**	2.57	1.3–5.0	<0.01	1.20	0.5–2.8	0.69
**ZEB2 Status (Pos vs. Neg)**	1.91fri	1.2–3.2	<0.01	1.9	1.6–6.6	<0.01

© 2018 Sreekumar R et al. *JAMA Network Open.*

eFigure 4

ZEB2 is an Independent Prognostic Marker of Survival in Patients with Non-Metastatic Rectal Cancer (Sub-Group Analysis)

Analysing the association between ZEB2 expression and oncological outcomes with specificity to patients with rectal cancer from both training and validation cohorts (n=84), demonstrated a significant reduction in both overall and disease free survival. Of the 84 patients analysed, 31 were ZEB2 positive and 53 ZEB2 negative. 32 (38.0%) patients received neo-adjuvant chemoradiotherapy. No association was noted between ZEB2 status and receiving neoadjuvant therapy. Patients with rectal cancers expressing nuclear ZEB2 experienced a mean reduction of 32 months in OS (log rank, $p<0.05$, eFigure 4A) and 30 months in DFS (log rank, $p<0.002$, eFigure 4B). Subdividing DFS into distant and local recurrence highlighted a significant association between ZEB2 expression and distant (log rank, $p<0.05$) but not local recurrence (log rank, $p=0.1$). Multi-variable Cox-regression analysis which included T-stage, N-stage, differentiation, EMVI status, neo-adjuvant and adjuvant therapies highlighted ZEB2 as an independent prognostic marker of both OS (HR 3.6, 95% CI 1.4 – 8.9, $p=0.006$) and DFS (HR 3.9, 95% CI 1.8 – 13.7, $p=0.04$) with specificity to distant recurrence.

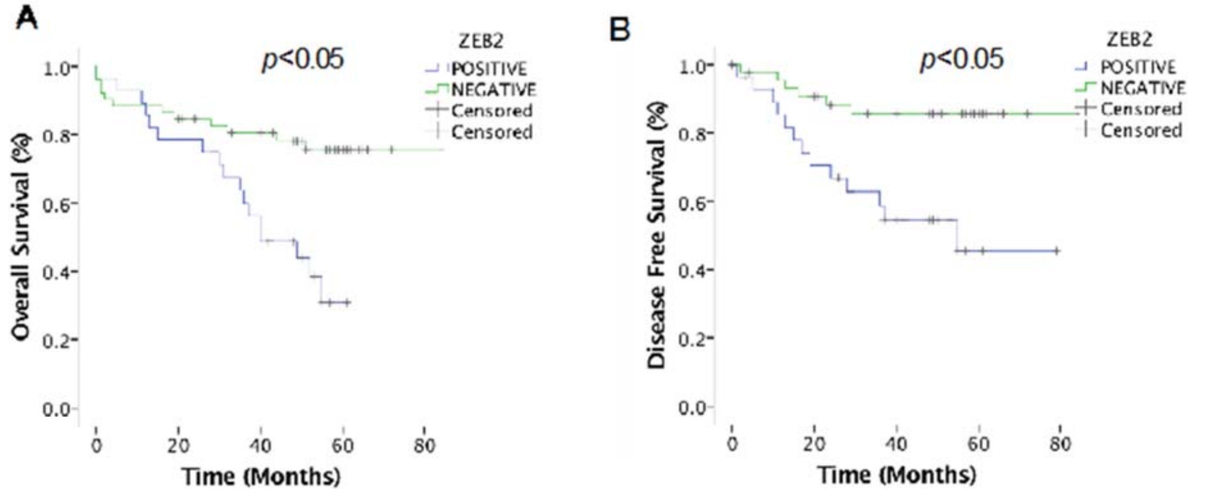

Months	0	20	40	60	80
ZEB2+VE No. at risk	31	22	14	2	0
Events	0	6	13	17	17
ZEB2-VE No. at risk	53	43	37	19	3
Events	0	8	10	12	12

Months	0	20	40	60	80
ZEB2+VE No. at risk	31	19	11	2	0
Events	0	8	12	13	13
ZEB2-VE No. at risk	53	37	31	15	3
Events	0	4	6	6	6

© 2018 Sreekumar R et al. *JAMA Network Open.*
